# Metabolic disorder of nutrients—an emerging field in the pathogenesis of preeclampsia

**DOI:** 10.3389/fnut.2025.1560610

**Published:** 2025-03-07

**Authors:** Shuyue Li, Jie Zhu, Ying Zhao, Ping An, Huanqiang Zhao, Yu Xiong

**Affiliations:** ^1^Obstetrics and Gynecology Hospital, Fudan University, Shanghai, China; ^2^The Shanghai Key Laboratory of Female Reproductive Endocrine-Related Diseases, Shanghai, China; ^3^Shenzhen Maternal and Child Health Hospital, Shenzhen, China

**Keywords:** preeclampsia, nutrient metabolism, pathogenesis, carbohydrate, lipid, amino acids

## Abstract

It is well acknowledged that metabolic disorder binds closely with preeclampsia, though some of the causal relationships are still ambiguous. This review systematically summarizes the metabolic characteristics of carbohydrates, lipids, amino acids, and glycans in preeclampsia, highlighting their roles in oxidative stress, trophoblast autophagy, inflammatory response, and vascular tone regulation. Key findings include upregulated glycolysis and impaired mitochondrial function contributing to ATP deficiency, dysregulated lipid metabolism exacerbating oxidative stress and vascular dysfunction, and amino acid imbalances disrupting immune responses and redox homeostasis. Emerging therapies, such as metformin and pravastatin, demonstrate potential in targeting these pathways for prevention and treatment. Here, we reviewed thoroughly the related literature with a view to delineating the potential association of nutrient metabolism with preeclampsia, so that we could explore a promising therapeutic approach.

## Highlights

- Metabolism of nutrients is identified as a field in the pathogenesis of preeclampsia.
- Preeclampsia undergoes metabolic reprogramming of nutrients.
- Metabolites interact between placental trophoblast and immune cells in preeclampsia.
- Metabolites can serve as potential prevention and treatment targets for preeclampsia.
- Validations on metabolites in prenatal diagnosis of preeclampsia are expected.

## 1 Introduction

Preeclampsia, a serious but mysterious pregnancy disorder syndrome, is characterized by newly-onset hypertension which occurs at or after 20-week gestation and terminal organ dysfunction in the form of proteinuria, acute kidney injury, liver dysfunction, eclampsia, and even death (1, 2). Specific to pregnancy, preeclampsia has an incidence of approximately 3–5%, characterized by preterm birth of 15% and maternal death of 42% (3). The problem is that there is a lack of effective interventions, since the exact mechanism of preeclampsia is unknown; once preeclampsia occurs, only the termination of pregnancy can relieve the symptoms. Therefore, it is imperative that research be conducted on the mechanism of preeclampsia, which plays a significant role in its prevention and treatment, thereby significantly reducing perinatal adverse outcomes.

Increasingly recognized as a metabolic disease, preeclampsia can be explained by its increased susceptibility in pregnant women with higher body mass index (BMI) (4). Studies have shown that metabolic reprogramming in preeclampsia involves significant alterations in nutrient metabolism. For example, impaired placental mitochondrial function and upregulated glycolysis result in ATP deficiency and oxidative stress, contributing to disease progression (5). Additionally, dysregulated lipid metabolism, characterized by elevated fatty acid levels and lipid peroxidation, has been linked to vascular dysfunction and endothelial damage (6). Though the mechanism of preeclampsia is scarcely understood, a consensus has been reached that it is attributed to oxidative stress, trophoblast cell autophagy, systematic inflammation, platelet aggregation and increased vascular tone (7–9). Thus, the metabolic changes that are involved in the occurrence and development of preeclampsia are worthy of due attention in the clinic.

Metabolism is a general term for a series of chemical reactions that occur in living organisms which need to sustain life. Since metabolism is a relatively macro concept, we mainly focused on the metabolism of nutrients such as carbohydrates, lipids, amino acids and glycans, which have disparate metabolic pathways to be connected through certain intermediate metabolites, so as to generate energy and synthesize substances that maintain normal functions of cells. In the field of oncology, up to now, the studies on metabolism catch the spotlight of the public, where researchers have found that metabolic reprogramming in tumor cells can help their proliferation by despoiling nutrients from the microenvironment (10). In the case of preeclampsia, currently, the metabolic investigations center on placental energy and metabolic intermediates, the alterations of which are caused by metabolic reprogramming, ultimately leading to insufficient placental energy and perturbation of nutrient synthesis. Although much research has been performed in this field, a systematic summary has not been made on the overall metabolic changes in preeclampsia. In the current review, we outlined the metabolic characteristics of nutrients systematically and completely, based on which we proposed some metabolism-related predictors and therapies for preeclampsia (11).

A comprehensive literature search was conducted using PubMed, Web of Science, and Scopus databases. The search included articles published in English up to 2023, using keywords such as “preeclampsia,” “nutrient metabolism,” “carbohydrates,” “lipids,” “amino acids,” and “glycans.” Inclusion criteria were peer-reviewed articles that investigated metabolic mechanisms or therapeutic approaches related to preeclampsia. Exclusion criteria included studies unrelated to nutrient metabolism or lacking experimental/clinical data.

## 2 Metabolic features of preeclampsia

**Figure 1:**
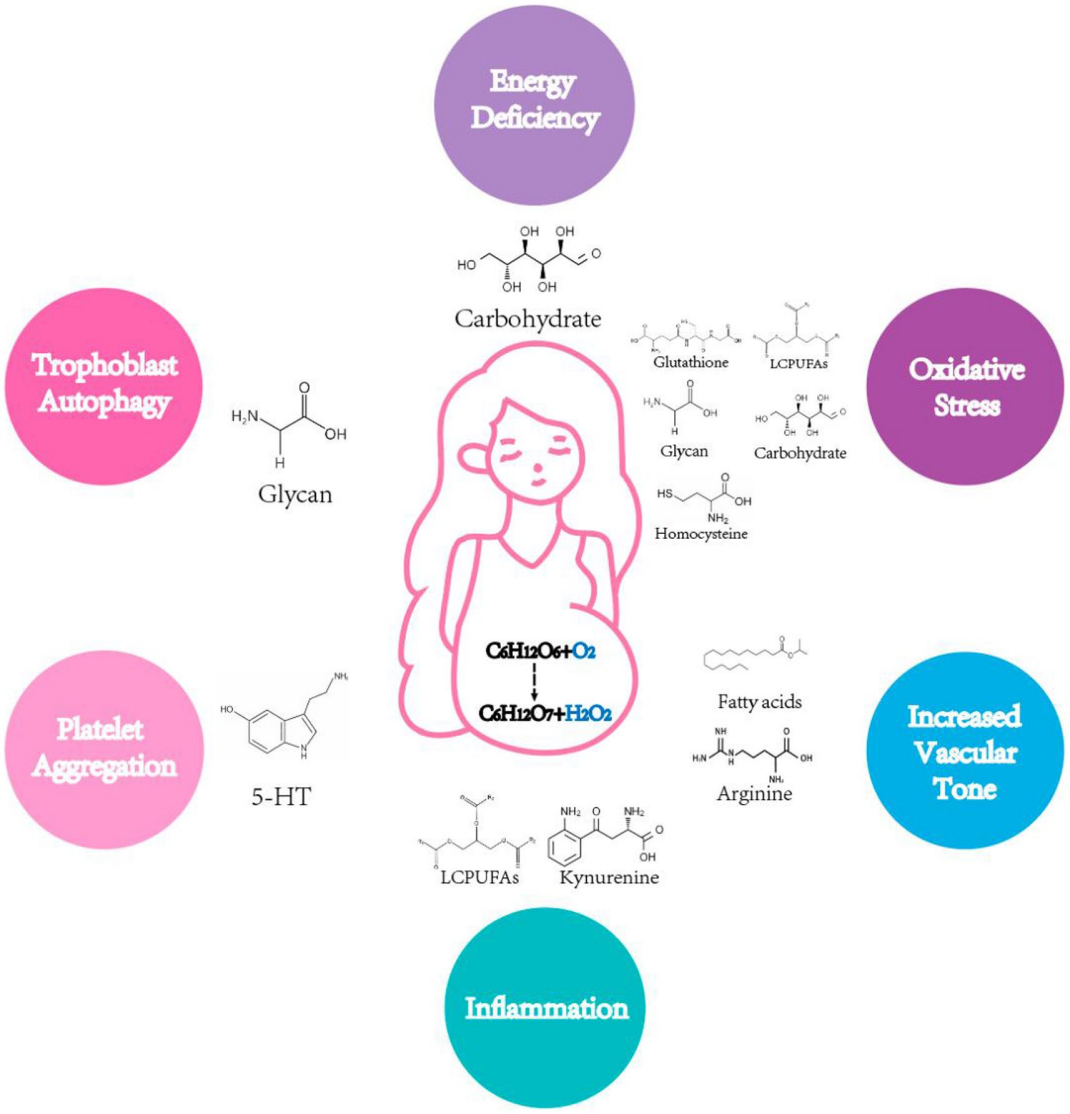
Pathogeneesis of preeclampsia regulated by nutrients metabolism. Several classic mechanisms of preeclampsia including energy deficiency, oxidative stress, increased vascular tone, inflammation, platelet aggregation and trophoblast autophagy can be regulated by metabolites.

The nutrients that can regulate the development of preeclampsia include carbohydrates, lipids, amino acids and glycans, which can have a principal effect on placental energy supply, inflammatory response, vasoconstriction, oxidative stress, trophoblast autophagy and vascular tone. As indicated in Figure 1, a category has been made of different nutrients and their metabolites, which may contribute to or inhibit the progression of preeclampsia through certain pathways.

### 2.1 Carbohydrates

**Figure 2:**
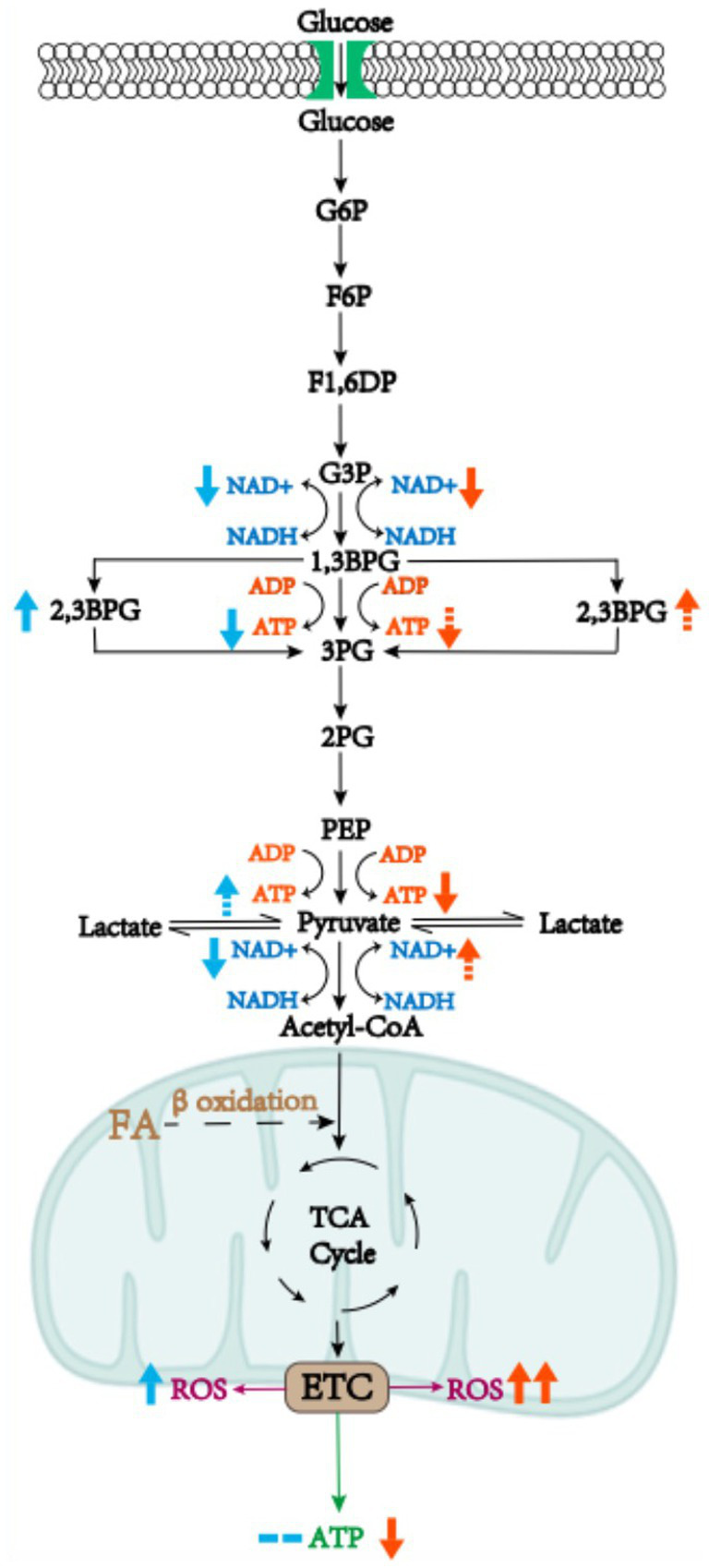
Metabolic reprogramming of carbohydrates in mild or severe preeclamptic placenta. In preeclampsia, the glycolytic pathway is activated and large amounts of NAD+ are consumed, leading to a blockage in the synthesis of vital substances in the placenta. Moreover, some of the ATP synthesis pathways are bypassed, ending up with less ATP production. Metabolic reprogramming of carbohydrates in mild preeclampsia compensates by increasing glycolytic intermediates, such as increasing oxidation of fatty acids to provide acetyl-coA, whereas in severe preeclampsia a decompensation of metabolic reprogramming of carbohydrates has been witnessed. The blue arrows represent the direction of metabolic reprogramming in mild preeclampsia and the red arrows represent decompensation of metabolic reprogramming in severe preeclampsia. Solid arrows represent changes that have been reported in the literature, dotted arrows represent presumed changes, and the number of arrows is proportional to the magnitude of the changes.

Carbohydrates, as organic compounds composed of carbon, hydrogen and oxygen, exist mostly in nature, with a broad spectrum of chemical structure and biological function. The most vital metabolic pathway of carbohydrates is central carbon metabolism involving glycolysis, pentose phosphate and tricarboxylic acid cycle, as the main source of energy for organisms and also as precursor for other metabolisms in the human body (12). As early as 1987, researchers observed reduced adenosine triphosphate (ATP) production in preeclamptic placenta, which was caused by placental ischemia and hypoxia, thus impairing energy-dependent placental functions such as active transport, i.e., amino acids transfer, and protein synthesis; at this point, glycolysis was upregulated to maintain ATP synthesis (13, 14). This could be explained by the changes in metabolic enzyme activity, which is the researchers’ initial understanding of carbohydrate metabolic reprogramming. Recent research provided a deeper understanding of this notion, revealing that carbohydrate metabolism could adapt to the hypoxia condition in mild preeclampsia while a decompensation of carbohydrate metabolism reprogramming was discovered in severe preeclampsia (11) (Figure 2).

In cells, glycolysis produces a small amount of ATP, while most of energy is produced via oxidative phosphorylation in mitochondria. Mitochondrial dysfunction and oxidative stress, manifested as the increased production of reactive oxygen species (ROS), are known to occur in different subtypes of preeclampsia (15). In mild preeclampsia, mitochondria may compensate by enhancing oxidative phosphorylation and antioxidant activity, and the glycolytic pathway is also compensatively activated to replenish ATP (16). Moreover, it has been demonstrated in mouse models that the levels of 2,3-Bisphosphoglycerate (2,3-BPG) derived from 1,3-Bisphosphoglycerate (1,3-BPG) are significantly increased, which bypassed ATP-producing pathway where 1–3 BPG synthesizes 3-Bisphosphoglycerate (3-BPG) (5). Activation of glycolysis is known to lead to a lack of NAD+, resulting in a decrease in the conversion from pyruvate to acetyl-CoA where NAD+ act as a coenzyme of pyruvate dehydrogenase. Tricarboxylic acid (TCA) cycle, another energy-supplying process buttressed by NAD+ as coenzyme, cannot proceed smoothly. Acetyl-CoA also has a compensatory pathway, coming from fatty acid β oxidation in addition to glycolysis (17). β oxidation of fatty acids is likely to produce acetyl-CoA as compensation, which can be demonstrated by increased acylcarnitine levels (5). Therefore, we hypothesize that in energy production, a compensatory increase in ATP production from glycolysis combined with mitochondrial oxidative phosphorylation could maintain overall ATP levels in mild preeclampsia, and that in terms of substance synthesis, the substances that need to maintain the normal function of the placenta can be ensured, thanks to the compensatory production of acetyl-CoA.

In the case of severe preeclampsia, the bypass pathway from 1,3-BPG to 2,3-BPG could also be activated, as it brought about ATP deficiency (5), a major character of preeclampsia. Mitochondrial dysfunction could not be compensated by increasing oxidative phosphorylation, so that it was only compensated by activating the glycolytic pathway, which was inefficient at producing ATP (18). A loss of bioenergy and biosynthetic homeostasis was reported, which could be explained by significantly reduced concentrations of intermediate metabolites such as pyruvate, lactic acid, and pyruvate kinase (a key enzyme in glycolysis) in placenta (16). Thus, we propose a hypothesis that the former part of glycolysis is activated in severe preeclampsia, but bypass the ATP-producing pathway, while the latter part is inhibited, resulting in decreased ATP production in glycolysis along with overall ATP decrease; this process in turn could lead to the clinical manifestation of severe preeclampsia, which comprised premature birth and fetal growth restriction (11).

### 2.2 Lipids

To meet the needs of fetal growth and development in normal pregnancy, the capacity of intestinal lipid absorption is enhanced (19–21); the elevated levels of blood lipid (such as cholesterol and triglycerides) lead to increased lipid peroxidation (22, 23). In contrast, women with preeclampsia are characterized by higher BMI, higher levels of blood lipid and lipid peroxides (24), which can be decomposed into more free radicals, thus damaging vascular endothelial cells, causing the decrease of prostacyclin (PGI2) by inhibiting PGI2 synthetase and activating thromboxin (TXA2) synthetase to produce TXA2 (25). PGI2/TXA2 ratio thereafter decreases, leading to a series of pathophysiological changes of vasospasm and contraction. Furthermore, free radicals could cause mitochondrial damages, followed by reduced energy production, and increased oxidative stress (6, 26). The free radicals induced by oxidative stress actively interact with polyunsaturated fatty acids (PUFAs) to produce lipid peroxides, forming a vicious cycle (27). Once confirmed in preeclampsia, the altered lipid metabolism is likely to be the result of genetic predisposition, which also acts as a risk factor for the development of cardiovascular disease in later life.

Although the effect of abnormal lipid metabolism on the classical mechanisms of preeclampsia has been well documented, the mechanisms of the various abnormalities in lipid metabolism have not been clearly elucidated, except for that of fatty acids, the important metabolites of fat which are increased in the second and third trimesters in normal pregnancy (28). Significantly elevated fatty acids is usually seen in preeclampsia, which can be observed prior to the onset of the disease (29), and attributed to decreased activity of corresponding metabolic enzymes (30). Gene mutation and polymorphism of short-chain acyl-coenzyme A dehydrogenase (SCAD, a type of enzyme in charge of fatty acid β oxidation), to name a few, are known to relate to the decreased activity of SCAD, thus raising the level of short-chain fatty acids, which acts as a risk factor in preeclampsia (30). In addition to alternation in genes, disturbance of intestinal flora can also leads to the reduction of fatty acid levels in the intestine and blood, which is related to the occurrence of preeclampsia because fewer fatty acid cracking products are found in the feces of pregnant women with preeclampsia (31). Therefore, the role of fatty acids in preeclampsia needs further study.

Additionally, the disorder of long chain fatty acid oxidation has been found in preeclampsia, which is characterized by the reduced level of mRNA and protein expression of long chain omega-3 hydroxy CoA dehydrogenase (LCHAD) in the placenta of preeclampsia, which leads to lipid deposition. The earlier the onset of preeclampsia, the more significant abnormal LCHAD expression and the more lipid deposition can be found (32). The altered activity of SCAD and LCHAD working together could lead to the overloaded titers of fatty acid in the serum, which could contribute to oxidative stress and increased vascular tone through the pathways aforementioned (33). The notion of SCAD deficiency has been validated based on a single clinical case; therefore, it is important that a large-sample-based analysis be conducted to search for the gene-induced fatty acid enzymes to induce irregularities in preeclampsia (30), and that future studies focus on the enzymes related to fatty acid metabolism and the gene expressions in the activity regulation of enzymes so as to prevent the occurrence and development of preeclampsia.

Beyond fatty acids, recent studies have highlighted the role of metabolic reprogramming in other major lipid classes, such as glycerophospholipids, sphingolipids, and long-chain polyunsaturated fatty acids (LCPUFAs), in the pathophysiology of preeclampsia (34). For instance, sphingolipids, particularly ceramides, have been implicated in trophoblast dysfunction and apoptosis, leading to impaired spiral artery remodeling, a hallmark of early-onset preeclampsia (35). Similarly, altered glycerophospholipid metabolism disrupts endothelial function and inflammatory signaling pathways, exacerbating vascular dysfunction (36). LCPUFAs, such as docosahexaenoic acid (DHA) and arachidonic acid (AA), are critical for maintaining vascular tone and regulating inflammation. Disruptions in the DHA/AA balance have been linked to increased oxidative stress and endothelial damage in preeclampsia (37).

### 2.3 Amino acids

**Figure 3:**
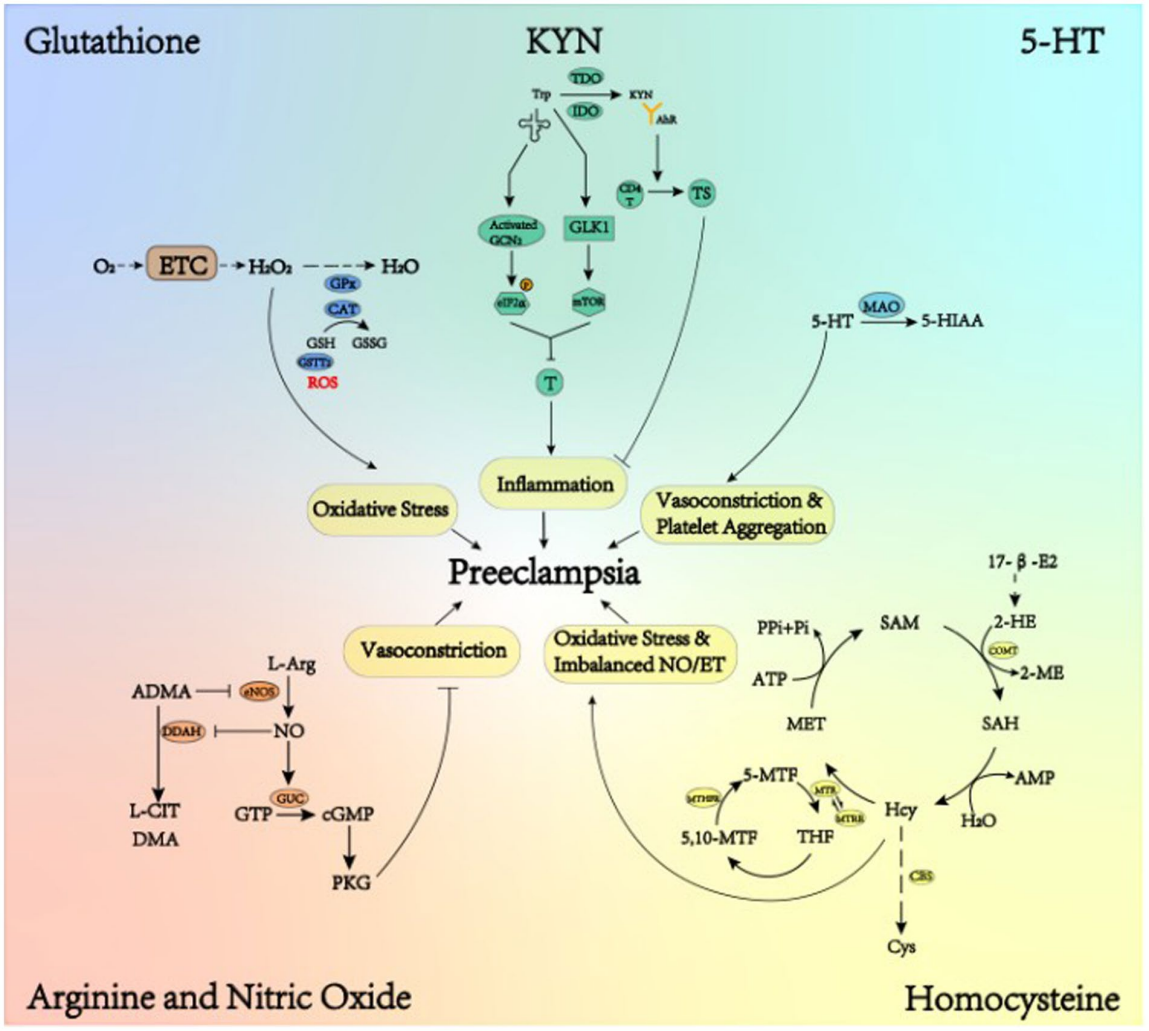
Metabolic pathways of various animo acid and their contributions to preeclampsia. In normal placental blood vessels, a variety of amino acids and their metabolites can regulate oxidative stress, vasoconstriction, inflammation and so on. In preeclamptic placentas, changes in the activity of enzymes that metabolize certain amino acid can lead to changes in the level of corresponding amino acid, thus having an impact on the above pathways. T, T cells; CD4T, CD4T cells; TS, inhibitory T cells.

The metabolism of the multiple varieties of amino acids, peptides and proteins have been newly proposed to correlate with the development of preeclampsia. As indicated in Figure 3, a focus has been made on glutathione (GSH), tryptophan (Trp), arginine (Arg) and homocysteine (Hcy) metabolism, which involve in the classical mechanism of preeclampsia, respectively.

#### 2.3.1 GSH

It has been experimentally verified that preeclampsia is responsible for oxidative stress, and that GSH is a prominent component of the non-enzymatic oxidative defense system (38). GSH is a major intracellular antioxidant compound, abundant in the cytoplasm, nucleus and mitochondria (39). Capable of bioconversion and elimination of biomass, GSH plays an antioxidant role when oxidized to glutathione oxidized (GSSG) through the catalysis of glutathione peroxidase (GPx) and catalase (CAT) (40). Moreover, GSH contributes to the conversion from H_2_O_2_ to H_2_O, thus alleviating oxidative stress where H_2_O_2_ acts as a vital type of ROS (41).

The higher level of GPx in the umbilical cord of preeclampsia has been observed, which may function as a compensatory mechanism against oxidative stress (21). This could explain the similar levels of H_2_O_2_ in preeclampsia in comparison with normal pregnancies (42, 43). Though the higher level of GPx has been found in preeclampsia, the changing activities of CAT remain controversial. It has been suggested that CAT is compensatively increased in preeclampsia (43), or that decreased CAT increases the risk of preeclampsia (44).

Additionally, the decreased expression of Glutathione S-transferase theta 2 (GSTT2) has been found in preeclampsia, which is a major type of enzyme in charge of combining ROS with GSH, thus reducing oxidative stress (45). In general, the decompensation of the aforementioned enzymes inhibits the scavenging of ROS, which is the root cause of redox imbalance in favor of pro-oxidants (43). In view of a considerable variety of enzymes involved in the metabolism of GSH, their changing activities in redox reaction and their potential roles in the prediction and therapeutic significance in preeclampsia can be of an intriguing discipline to pursue.

#### 2.3.2 Trp

Trp, an essential amino acid in the human body, is known to have two breakdown pathways: the kynurenine (KYN) and the 5-HT pathway. It was previously reported that a small part of Trp produced 5-HT through tryptophan hydroxylase (TPH), whereas about 95% of Trp produced KYN through the catalysis of indolamine 2,3-dioxygenase (IDO) or tryptophan2,3-dioxygenase (TDO) (46).

##### 2.3.2.1 KYN

By depleting Trp, IDO is known to inhibit the function of immune cells, including T lymphocyte proliferation, and to potentially downregulate the inflammatory response. IDO inhibits the function of immune cells through a specific L-Trp depletion pathway, where L-Trp depletion by IDO leads to its dissociation with L-Trp tRNA, thus activating general control non-derepressible 2 (GCN2) due to the presence of its allosteric regulatory site which senses free tRNAs (47, 48). Phosphorylated by activated GCN2, the activity of eukaryotic translation initiation factor-2α (eIF-2α) is weakened, thus inhibiting the transcription of various RNAs and the translation of proteins in T cells (27). Moreover, L-Trp deficiency can inhibit master amino acid-sensing kinase 1 (GLK1), and further inhibit m-TOR signaling molecules, triggering T cell impotence and autophagy (27). Additionally, KYN produced by IDO is an endogenous ligand of aromatic hydrocarbon receptor (AhR). KYN binding to AhR leads to differentiation of immature CD4+ T cells into inhibitory T cells; it can also induce IDO expression, further suppressing T cell immune response and inflammation (49).

In normal pregnancy, plasma KYN/Trp ratio increases with gestational age. In preeclampsia, however, a decrease in plasma KYN/Trp ratio has been discovered, which can be attributed to decreased placental IDO activity (50). IDO-caused failure of Trp depletion was reported to induce excessive inflammatory response in preeclampsia (50). On the other hand, a significant increase in TDO expression has been found in preeclampsia, but since inflammatory response is involved in the pathogenesis of preeclampsia, it can be hypothesized that upregulation of TDO cannot fully compensate for the decrease in IDO in this regard. It is important that future studies address the differential regulation of IDO and TDO in placenta to determine their mechanistic role in preeclampsia (51, 52).

##### 2.3.2.2 5-HT

As a metabolite of Trp, 5-HT has been described primarily as a potent vasoconstrictor in the placental circulation (52). 5-HT activates its receptors on vascular smooth muscle and platelets, thereby promoting vasoconstriction and platelet aggregation. Furthermore, 5-HT synergistically amplifies the effects of other vasoconstricting substances (53). Catalyzed by monoamine oxidase (MAO), 5-HT metabolizes into 5-hydroxyindole acetic acid (5-HIAA), which is excreted from the body in the form of urine (54).

Women with preeclampsia have less 5-HIAA excreted from the urine, the evidence that cannot be explained in terms of impaired renal function, which is one of the manifestations in preeclampsia (55). At the same time, the patients’ level of 5-HT is high (40). The increased serum concentration of 5-HT and the simultaneously decreased excretion of 5-HIAA can be attributed to the decreased activity of MAO (50, 54, 56). Increased 5-HT is known to exacerbate the effect of vasoconstriction and platelet aggregation. One piece of evidence has shown that the involvement of increased 5-HT in the pathogenesis of preeclampsia could be the therapeutic effect of ketanserin, a 5-HT2 antagonist (53). Ketanserin functions as antihypertensive and antithrombotic, improving hemodynamics (57). Experimental 5-HT infusion in the pregnant animal could cause the similar kidney and placental damages as those seen in preeclampsia, which can be prevented by ketanserin (50). Even though less is known about the reason behind the decreased expression of MAO, MAO activity can determine, partially at least, the maternal systemic 5-HT concentration.

#### 2.3.3 Arg and nitric oxide (NO)

Arg, a non-essential amino acid produced at a slow speed, plays an irreplaceable role in the human body as a basic component of various proteins. There exist three Arg metabolic pathways, of which the one producing NO under the catalysis of nitric oxide synthase (NOS) is of the most significance. NO is known to serve as a strong vasodilator, playing an effective role in maintaining the constancy of vascular tone and regulating the stability of blood pressure (58, 59). Studies have shown that NO affects cardiovascular system mainly by activating guanylate cyclase (GUC) to increase the concentration of guanosine cyclomonophosphate (cGMP) (60–65). As a second messenger, cGMP mediates the inhibition of calcium ion flow through receptor-mediated calcium channels (53). Activation of GUC mediated by NO completes various physiological functions such as vasodilation and inhibition of platelet aggregation (66).

In preeclamptic placentas, reduced L-Arg and NO formation contributes to the inactivation of GUC-related channel, which may be attributed to the increased level of asymmetric dimethylarginine (ADMA), down-regulated eNOS and dimethylarginine dimethylamine hydrolase (DDAH) expression respectively (2, 67). NO produced by eNOS is downregulated through ADMA-caused reversible competitive inhibition (68). Since ADMA competes with Arg for eNOS, the bioavailability of NO depends on the balance between Arg and ADMA, namely the Arg/ADMA ratio; in preeclampsia the lower Arg/ADMA ratio contributes to less production of NO (69), yet eNOS do not differ significantly between the early-onset and late-onset of preeclampsia (70). ADMA itself is metabolized to L-citrulline and dimethylamine by DDAH, whose reduced activity in preeclampsia can result in increased ADMA level, which competes with eNOS for NO and reduces NO production (58). The contributing factors aforementioned can cause the overall level of NO to decrease, thus inactivating GUC-related channel, hence the platelet aggregation and high blood pressure ultimately in preeclampsia (71).

According to other researches, nonetheless, women with preeclampsia tend to increase the major metabolite of NO in their serum and urine, mainly in the form of FeNO (72, 73). Moreover, placental eNOS activity is not significantly different between preeclampsia and normal pregnancies (74). Evidence has shown that the changes in placental NO metabolism are unlikely to be the main cause of placental lesions, that the higher level of circulating NO metabolites may be to compensate for the vasoconstrictor effect of preeclampsia, and that the vascular system of patients with preeclampsia may also have some degree of desensitization or resistance to the effects of NO (74). Therefore, the exact changes of NO in preeclampsia remain to be further investigated.

#### 2.3.4 Hcy

Hcy, a member of the methionine-homocysteine metabolism (MHM), is transformed into methionine (MET) (75) by catalyzing methyltransferase (MTR), which uses 5-methyltetrahydrofolate (5-MTHF) as a methyl donor (76). 5-methyltetrahydrofolate-homocysteine methyltransferase reductase (MTRR) is capable of regenerating functionally active MTR by reducing methylation. 5,10-methylenetetrahydrofolate reductase (MTHFR) provides methyl groups with tetrahydrogen folic acid (THF) into 5-MTHF so that MHM receives methyl groups. Using ATP as an adenosine donor, MET is transformed into S-adenosylhomocysteine (SAM), which gets rid of a methyl group to transform SAM, which goes through the process of deadenylation and changes into Hcy. Catalyzed by cystathionine β synthase (CBS), Hcy turns into cysteine (Cys) (77).

In the normal pregnant women, plasma concentration of Hcy is low during the first trimester, which reaches its lowest level during the second half of pregnancy (78). In those who were diagnosed with preeclampsia; however, the level is increased, with hyper-homocysteinemia arising from MHM to cause oxidative stress and imbalance of plasma NO/ET level. In preeclampsia, hyper-homocysteinemia could be ascribed to the alternations of MTHFR and CBS, which, from the perspective of gene, are likely to be induced by single nucleotide polymorphisms (75). Since MTHFR is a polymorphic enzyme (79), the preeclamptic women are broadly observed to be homozygous for MTHFR T/T, MTHFR C677T and A1298C (80), which thus can lead to an increase in Hcy level. When the lower mRNA expression of CBS is found in preeclamptic placenta, moreover, a failure may occur in the elimination of Hcy (81).

Even though the changes in single nucleotide polymorphisms of MHM enzymes can lead to increased Hcy, MHM can be compensatorily activated in preeclampsia. 2-methoxyestradiol (2-ME), a metabolite of 17-𝛽-estradiol synthesized by Catechol-O-Methyltransferase (COMT), induces the differentiation of the endovascular cytotrophoblast cells into its invasive phenotype under the condition of hypoxia (75). With COMT being responsible for methylating 2-Hydroxyestradiol (2-HE) into 2-ME, it has been demonstrated that low activity or expression of this enzyme could be involved in the pathogenesis of preeclampsia (82). MHM, the process responsible for supplying COMT with the methyl group necessary for 2-ME synthesis, could be compensatorily activated in preeclampsia to supply methyl groups enough to sustain adequate concentration of 2-ME (83).

The notion that MHM is activated as compensation can be justified by the post-transcriptional changes of the related enzymes in MHM. In the preeclamptic placentas, RNA expression of MTHFR and MTR is elevated, but this change is not reflected in protein content, which highlights a potential compensatory mechanism for MHM (75). This underlines a possible role of MHM as a compensation mechanism in the presence of low 2-ME levels (83).

#### 2.3.5 Changes of amino acids in severe preeclampsia

**Figure 4:**
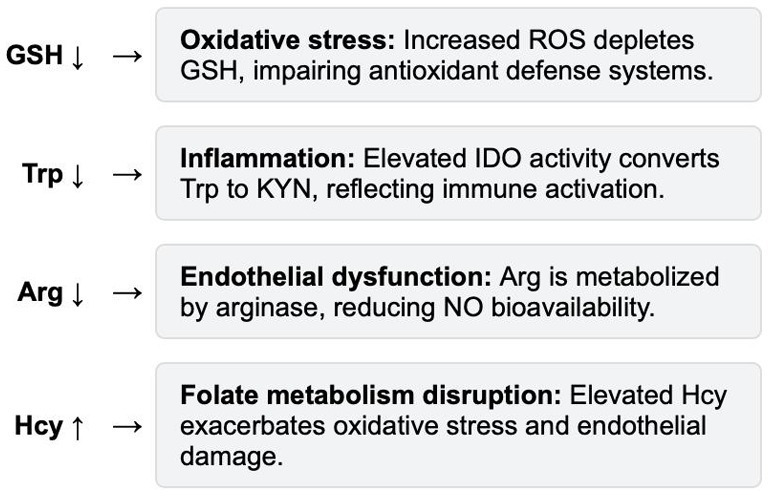
Key amino acid changes and mechanisms in sPE.

In severe preeclampsia (sPE), oxidative stress, inflammatory responses, and endothelial dysfunction collectively contribute to significant alterations in various metabolic molecules. GSH, as an important endogenous antioxidant, is significantly depleted due to elevated oxidative stress, leading to impaired antioxidant defense systems (84, 85). Additionally, the level of Trp decreases as a result of increased activity of IDO, which metabolizes Trp into KYN, reflecting enhanced inflammation and immune activation (86). Arg levels are reduced in sPE, primarily due to endothelial dysfunction. Arg, as a substrate for NO synthesis, is metabolized by arginase into ornithine and urea, further reducing NO bioavailability (87, 88). Additionally, Hcy levels increase due to disrupted folate metabolism or deficiencies in vitamins B6/B12. The accumulation of Hcy exacerbates oxidative stress and endothelial dysfunction, promoting the progression of preeclampsia (89, 90). These amino acid alterations in sPE illustrate the intricate interplay between oxidative stress, inflammation, and endothelial dysfunction, providing valuable insights into the disease’s pathophysiology. Figure 4 summarizes the key changes and their associated mechanisms.

### 2.4 Glycan

Glycan, a complex composed of monosaccharides, can covalently bind with proteins or lipids, forming such biomolecules as glycoproteins, proteoglycans and glycolipids. The complex is known to be involved in cell recognition, cell adhesion, cell differentiation, immune recognition, and even tumor metastasis. In the placenta, the expression of glycan is a dynamic reflection of placental developmental and pathophysiological state. In the case of preeclampsia, oxidative stress can lead to the excessive production of ROS, which can regulate the expression of glycan (26). The higher expression of mannosan, a subtype of glycan, can be found at the end of placental villi in the early-onset of severe preeclampsia, and it can be recognized by cytotoxic NK cell mannose-receptors, which activate NK cells to make systemic inflammatory responses (91). In addition to preeclampsia, glycation of trophoblastic cells is also associated with pregnancy-induced hypertension and fetal growth restriction (92).

As an important “interpreter” of glycan, galectin can bind with glycan to act as an “alarm protein-like” molecule signalizing tissue damages induced by oxidative stress (91). Since apoptosis results partially from oxidative stress, the galectin-glycan circuit acts indispensably in regulating pro-survival and pro-apoptosis pathways, maintaining homeostasis under microenvironmental damages (92). The changes in the glycan of placental villous, caused by excessive ROS, can lead to the decreased level of Gal-9, a type of galectins most expressed at the maternal fetal interface, whose increased levels indicate that the placental cells are susceptible to apoptosis (93). Autophagy exists in normal human placenta and is an important manifestation of placenta’s normal physiological function. Apoptosis is found in normal placental trophoblast, stromal and endothelial cells, but mainly in syncytiotrophoblast (94). It has been confirmed in animal models that the placenta of mice with autophagy deficiency shows typical pathological changes of preeclampsia, that is, superficial trophoblast invasion and vascular remodeling failure, affecting the normal pregnancy process (95). Given that apoptosis and autophagy are mutually inhibited, apoptosis facilitates Gal-9-mediated autophagy reduction, which results in the decrease of invasiveness in the trophoblastic cells, and the obstruction of spiral artery recasting, hence placental hypoperfusion (96). Therefore, the dynamic changes in the galectin-glycan network associated with oxidative stress may play an important role in the pathogenesis of preeclampsia. This local stress response can eventually spread throughout the body, resulting in the development of preeclampsia (92).

However, the effect of glycosylation on autophagy and reactive immune cell regulation in the trophoblast requires further investigation, as excessive oxidative stress has been found to reduce the expression of GnT-III, a key mannose glycosyl transferase, contrary to the previously stated hyper glycan expression in the terminal of the placental villus in preeclampsia (97). This may be due to different cell lines in different experiments. In conclusion, oxidative stress-induced glycosylation of the placental trophoblast cells can change their metabolic traits, exerting an impact on the development of preeclampsia.

## 3 Immune cells regulated by nutrient metabolism in preeclampsia

Interaction exists between immune cells and trophoblast, as manifested by the evidence that CD8 + T cells induce trophoblast to express matrix metalloproteinase-2/9 (MMP-2/9) to promote trophoblast invasion and facilitate embryo implantation. As aforementioned, the high concentration of fatty acid is observed in the preeclamptic placenta, as a risk factor. Even though the role of fatty acid in the interaction between immune cells and trophoblast cells remains obscure, it can be referred to as the microenvironment of tumor metabolism, as tumor and trophoblast cells share the common character of metabolic reprogramming. In the microenvironment of tumor metabolism, the higher levels of fatty acid inhibit CD8 + T cell function and promote tumor growth by altering the metabolic pattern of tumor cells (98). Tumor and CD8 + T cells appear to reprogram fatty acid metabolism differently, for the tumor cells adapt themselves by increasing fatty acid utilization, whereas CD8 + T cells do not (98). The uptake of fatty acids is enhanced by tumor cells, which may contribute to the deficiency of fatty acids in the tumor microenvironment of CD8 + T cells, whose normal function is impaired (98). Thus, the higher levels of fatty acid exacerbate metabolic reprogramming, which can lead to the nutrient availability and immune dysfunction altered in the tumor microenvironment.

Therefore, we hypothesize that in the preeclamptic placenta the high fatty acid concentrations can be responsible for metabolic reprogramming as in the case of tumor cells, resulting in insufficient fatty acid availability for CD8 + T cells and inhibited CD8 + T cell function. Decidual CD8 + T cells could recognize human leukocyte antigen (HLA)-C expressed by extravillous trophoblast cells (99). Recognition of HLA-C by CD8 + T cells may be important to normal pregnancy, especially when those who lack killer cell activating receptors to interact with HLA-C are more likely to suffer from preeclampsia (100). Recognition by decidual CD8 + T cells of HLA-C expressed by trophoblasts tend to result in the generation of IL8, which has been reported to increase production of both MMP-2 and MMP-9 by endothelial cells (101). This can be responsible for the vascular remodeling required for the establishment of the placenta (98). In other words, CD8 + T cell function, when inhibited, can impair trophoblast invasion, which is acknowledged as pathogenesis of preeclampsia. In view of this, it is imperative that the metabolite-mediated immune cell function and its interaction with the trophoblast cells be further studied in the future. Apart from the role of CD8 + T cell, other types of immune cells are likely to interfere with metabolites, which is an intriguing aspect of further investigation.

## 4 Treatments and preventions related to metabolism

**Figure 5:**
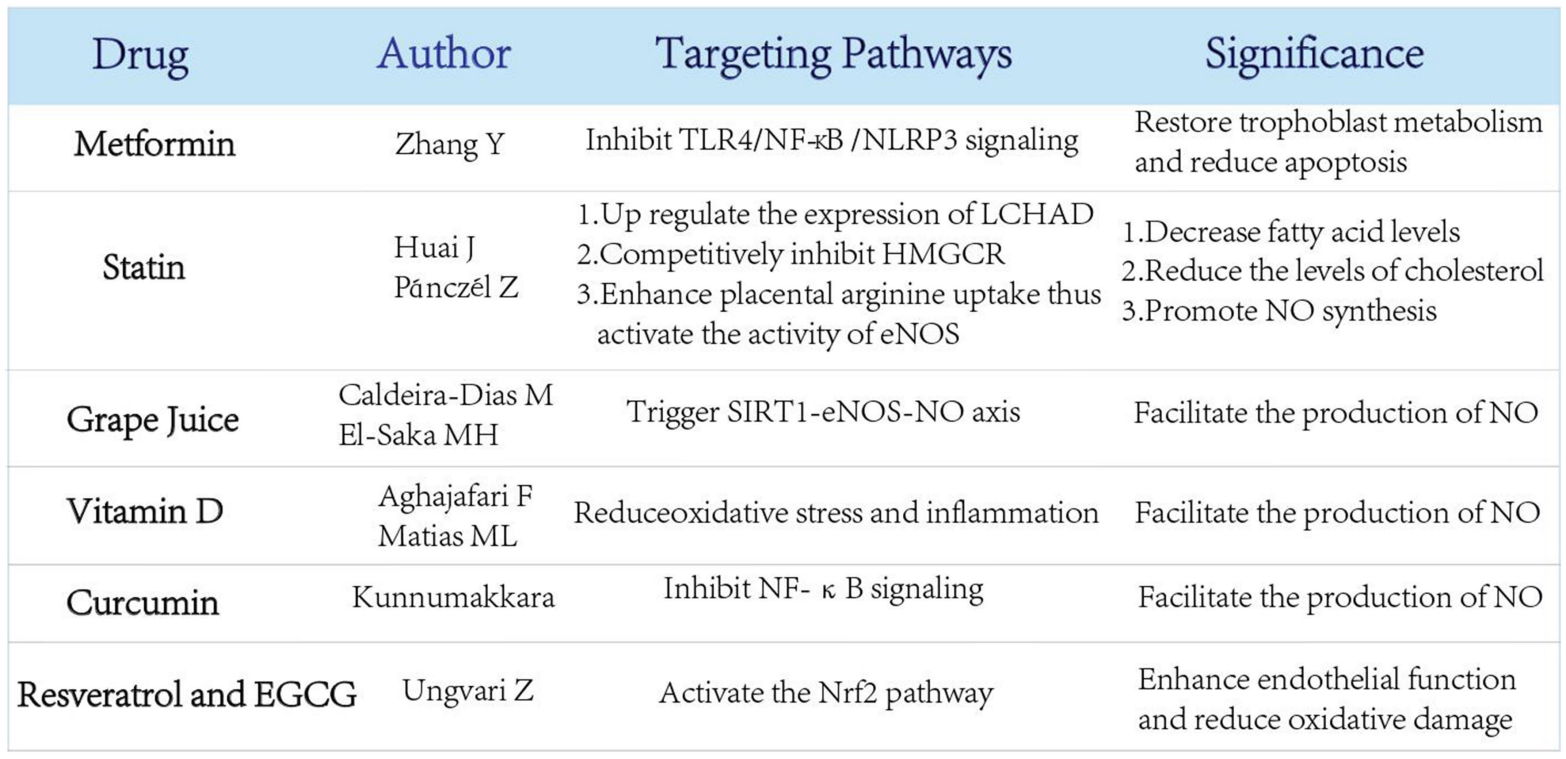
Several newly proposed therapies for preeclampsia related to nutrient metabolism and their potential effects. Several emerging treatments related to nutrient metabolism have been proposed to prevent the occurrence of preeclampsia through multiple regulatory pathways.

Although definitive therapies are unavailable, some metabolism-related ones have recently been shown to be promising in the prevention and treatment of preeclampsia by interfering with the classic mechanisms (Figure 5).

### 4.1 Metformin

Preeclampsia is thought to be associated with abnormal apoptosis of trophoblast cells. As recent studies have suggested that preeclamptic trophoblasts are highly likely to undergo glycolytic reprogramming, the newly discovered TLR4/NF-κB/PFKFB3 pathway may function as a link between metabolic reprogramming and NLRP3 inflammasome induced trophoblast apoptosis (102). In this pathway, TLR4/NF-κB signaling causes mitochondrial destruction and dysfunction, thus reprogramming the glycometabolism to glycolysis with increased PFKFB3 expression, which induces NLRP3 inflammasome assisted apoptosis (103–105). Activation of TLR4/NF-κB/PFKFB3 pathway in preeclampsia causes trophoblast cells to preferentially use glycolysis over mitochondrial oxidative phosphorylation, ultimately resulting in trophoblast ATP deficiency and increased apoptosis (102).

Metformin (MET), the first-line drug for type II diabetes mellitus, has long been clinically administered to regulate glucose metabolism. MET can reduce NLRP3-induced apoptosis and restore trophoblast metabolism by effectively inhibiting TLR4/NF-κB signaling (102, 106, 107). Beyond that, MET is partially capable of suppressing apoptosis by blocking the binding of the transcription factor NF-κB to PFKFB3 promoter and reducing PFKFB3 transcription (102).

Metformin is widely used during pregnancy for conditions such as gestational diabetes mellitus and polycystic ovary syndrome, with studies showing no significant increase in adverse outcomes for mothers or neonates. A Phase II clinical trial involving 180 pregnant women suggests that metformin (3 g daily dose) can extend the gestational weeks of early-onset preeclampsia by about 1 week and reduce neonatal hospital stay (108).

However, the long-term physiological effects of metformin therapy in pregnancy women without diabetes remains unclear till now. Therefore, MET and its long-term effect can have promising potential to be explored in preeclampsia.

### 4.2 Statin

Pravastatin, as a natural compound of statin, is conventionally applied to the treatment of primary hypercholesterolemia, type IIa and type IIb hyperlipidemia. In the case of preeclampsia, the level of LCHAD is found to be decreased, which is explained by the decreased level of fatty acid oxidation in this disease (102). Pravastatin comes in to play when the expression of LCHAD is upregulated in the liver and placenta, thus significantly decreasing fatty acid levels (109). Acting as a competitive inhibitor of 3-hydroxy-3-methylglutarate monoacyl-CoA reductase (HMGCR), the rate-limiting enzyme of cholesterol synthesis, pravastatin can also reduce the levels of cholesterol, low-density lipoprotein, very low-density lipoprotein and triacylglycerol in the body in a direct or indirect way, so as to regulate blood lipid level (110). Additionally, the potency of pravastatin can even be administered to regulate blood lipid so that the clinical manifestations of preeclampsia can be alleviated (111).

Apart from the lipid-regulating properties, pravastatin is capable of significantly increasing NOS activity in the placenta, thereby promoting NO synthesis (112). Preeclamptic pregnancies are known to have low concentrations of Arg in serum, where pravastatin induces Arg uptake at low Arg levels, rapidly activating eNOS whose activity increases with the supply of substrate (113). Future studies are acquired to explore the effect of pravastatin on the unknown levels of Arg in preeclamptic placentas and in severity-categorized preeclamptic samples.

### 4.3 Grape juice

As aforementioned, NO acts as a vasodilator in pregnancy. Since grape juice is found to interfere with NO production, a recent proposal has been made to use it as original add-on therapy for preeclampsia (114). Upon an ingestion of grape juice, NO production is increased in the serum endothelial cells of preeclampsia patients (115), which is recognized to trigger SIRT1-eNOS-NO axis. However, grape juice’s ability to increase the production of NO in endothelial cells does not appear to rely solely on its major antioxidant named resveratrol. In an *in vitro* PE model, grape juice intake appears to have a different effect than resveratrol supplementation alone, suggesting that other bioactive molecules in grape juice combined with SIRT1-eNOS-NO have therapeutic potential in PE (116). This suggests that grape juice can be of a feasible therapy for preeclampsia, although further experiments are required to measure its appropriate dose and other effective substances apart from resveratrol to better understand its synergistic effect along with SIRT1-eNOS-NO axis.

### 4.4 Vitamin D, natural compounds of plant origin and herbal extracts

Recent studies have highlighted the role of vitamin D and natural plant-derived compounds in reducing oxidative stress and inflammation, which are key contributors to preeclampsia pathogenesis. Vitamin D has been shown to regulate immune responses and improve endothelial function, with low maternal vitamin D levels being associated with an increased risk of preeclampsia (117). Supplementation with vitamin D has demonstrated potential in reducing preeclampsia risk in randomized controlled trials, although further large-scale studies are needed to confirm its efficacy (118).

Natural compounds of plant origin, including flavonoids, epigallocatechin gallate (EGCG), quercetin, resveratrol, and curcumin, have attracted attention for their antioxidant and anti-inflammatory properties. For example, curcumin has been reported to modulate oxidative stress pathways and inflammation through the inhibition of NF-κB signaling (119). Similarly, resveratrol and EGCG enhance endothelial function and reduce oxidative damage by activating the Nrf2 pathway (120). Quercetin, a potent flavonoid, has shown promise in preclinical studies for its ability to reduce vascular inflammation and improve placental function (121). These compounds may represent complementary therapeutic strategies, but more clinical studies are required to evaluate their safety and efficacy in pregnancy (122).

## 5 Clinical prospects and conclusions

In the pathogenesis of preeclampsia, the alternations of carbohydrates, lipids, amino acids and glycans are involved in the multiple classic mechanisms, the influence of which is significantly extensive. The main reason for the changes in metabolites, which still remains unclear though, is focused on the changes of relevant enzymes. However, the post-translational modification of proteins or other regulatory effects of metabolites have not been fully elucidated. Some newly proposed therapies which target nutrient metabolism have been shown promising in the prevention and treatment of preeclampsia in either animal models or patients. These findings reveal that metabolic abnormalities may involve in the pathophysiological mechanism of preeclampsia, which suggests that in the future researches, more specific metabolic pathways need to be explored in preeclampsia based on animals and *in vitro* models, which is of great significance to the development of new metabolic drugs for the alleviation of preeclamptic symptoms. Furthermore, large-scale cohort studies are urgently needed to validate the role of specific metabolites in prenatal diagnosis. These studies would help identify potential pathways and biomarkers critical to improving early detection and therapeutic strategies for preeclampsia.

## Glossary

BMI: body mass index
ATP: adenosine triphosphate
ROS: reactive oxygen species
2,3-BPG: 2,3-Bisphosphoglycerate
1,3-BPG: 1,3-Bisphosphoglycerate
3-BPG: 3-Bisphosphoglycerate
TCA: tricarboxylic acid
PGI2: prostacyclin
TXA2: thromboxin
PUFAs: polyunsaturated fatty acids
SCAD: short-chain acyl-coenzyme A dehydrogenase
LCPUFAs: long-chain polyunsaturated fatty acids
DHA: docosahexaenoic acid
AA: arachidonic acid
LCHAD: long chain omega-3 hydroxy CoA dehydrogenase
GSH: glutathione
Trp: tryptophan
Arg: arginine
Hcy: homocysteine
GSSG: glutathione oxidized
GPx: glutathione peroxidase
CAT: catalase
GSTT2: Glutathione S-transferase theta 2
KYN: kynurenine
TPH: tryptophan hydroxylase
IDO: indolamine 2,3-dioxygenase
TDO: tryptophan2,3-dioxygenase
GCN2: general control non-derepressible 2
eIF-2α: eukaryotic translation initiation factor-2α
GLK1: master amino acid-sensing kinase 1
AhR: aromatic hydrocarbon receptor
MAO: monoamine oxidase
5-HIAA: 5-hydroxyindole acetic acid
NO: nitric oxide
NOS: nitric oxide synthase
GUC: guanylate cyclase
cGMP: guanosine cyclomonophosphate
ADMA: asymmetric dimethylarginine
DDAH: dimethylarginine dimethylamine hydrolase
MHM: methionine-homocysteine metabolism
MET: methionine
MTR: methyltransferase
5-MTHF: 5-methyltetrahydrofolate
MTRR: 5-methyltetrahydrofolate-homocysteine methyltransferase reductase
MTHFR: 5,10-methylenetetrahydrofolate reductase
THF: tetrahydrogen folic acid
SAM: S-adenosylhomocysteine
CBS: cystathionine β synthase
Cys: cysteine
2-ME: 2-methoxyestradiol
COMT: Catechol-O-methyltransferase
2-HE: 2-Hydroxyestradiol
sPE: severe preeclampsia
MMP-2/9: matrix metalloproteinase-2/9
MET: metformin
HMGCR: 3-hydroxy-3-methylglutarate monoacyl-CoA reductase
EGCG: epigallocatechin gallate
